# Is it feasible and effective to provide osteopathy and acupuncture for patients with musculoskeletal problems in a GP setting? A service evaluation

**DOI:** 10.1186/1471-2296-12-49

**Published:** 2011-06-13

**Authors:** Anna Cheshire, Marie Polley, David Peters, Damien Ridge

**Affiliations:** 1 School of Life Sciences, University of Westminster, 115 New Cavendish Street, London, W1W 6UW UK; 2 Polyclinic, University of Westminster, 115 New Cavendish Street, London, W1W 6UW UK

## Abstract

**Background:**

Spinal manipulation and acupuncture can be helpful in reducing the symptoms of musculoskeletal (MSK) pain. Both approaches are currently recommended by NICE as treatment options for patients with persistent low back pain. However, there has been no previous evaluation of a GP service using them together for MSK pain. The purpose of this study was to evaluate acceptability and outcomes for an osteopathy and acupuncture service (delivered by complementary therapy practitioners) for patients with MSK problems provided within a General Practice.

**Methods:**

Patients were asked to complete a questionnaire before and after their course of treatment. Outcome measures included the Bournemouth Questionnaire (measuring MSK problems), EuroQoL-5D (measuring quality of life), medication use, physical activity and general well-being. Non-parametric tests were used to compare pre- and post- treatment variables. Qualitative data, regarding participants' views on the service, were collected from patients via a service survey and healthcare professionals via interviews. Qualitative data were analysed using thematic analysis.

**Results:**

123 adults with MSK problems were referred into the service (79 female and 44 male, mean age 49 years). Complete patient questionnaire data sets (pre- and post- treatment) were available for 102 participants; 91 completed a service survey. All healthcare professionals involved in the service participated in interviews including all seven GPs and the administration manager at the practice, as well as the three acupuncture/osteopathy practitioners.

Patient outcomes: comparisons between pre and post-treatment revealed a statistically significant improvement in MSK pain (p < 0.0001) and quality of life (p < 0.0001), and a statistically significant reduction in medication use (p < 0.0001). Qualitative analysis found that patients reported improvements in their MSK pain, mobility, other physical health conditions, well-being and self-management of their MSK problem.

Acceptability of the service: overall patients and healthcare professionals were satisfied with the service and its provision within the Practice. Patients reported wanting increased appointment availability and flexibility, and more sessions. Complementary therapy practitioners reported finding the high number of referrals of chronic patients challenging, and wanting increased communication with GPs.

**Conclusions:**

Provision of acupuncture and osteopathy for MSK pain is achievable in General Practice. A GP surgery can quickly adapt to incorporate complementary therapy provided key principles are followed.

## Background

Chronic pain currently affects 7.8 million people in the UK and it has been estimated that back pain alone costs the economy £12.3 billion per year [1]. A survey of adults registered with GPs in the UK found that 38% of respondents were affected by musculoskeletal (MSK) pain [2]. Dealing with MSK problems places a heavy burden on primary care services and resources [3]. However, treatment for MSK problems is perceived by GPs and other health professionals as an 'effectiveness gap' within the NHS [4,5]. Furthermore, the Chief Medical Officer's 2008 report recommends that much more needs to be done to improve outcomes for patients with pain, arguing that patient-centred services are essential, yet current systems and infrastructure are inadequate to meet patient needs and demand [1].

Despite NICE guidelines recommending manual therapy (which can be conducted by osteopaths) and acupuncture for persistent low back pain [6], there has been no previous evaluation of a GP practice using them together for this common problem. Yet there is good evidence for the potential benefits of providing osteopathy and acupuncture within primary care as treatment options for people with common MSK problems. Evidence from randomised controlled trials (RCTs), meta-analyses and a Cochrane review demonstrate that acupuncture can be useful in reducing lower back and neck pain [e.g. [7-10]], chronic shoulder pain [11], chronic knee pain [12] and reducing symptoms of knee and hip osteoarthritis [e.g. [13-15]]. RCTs have also shown that osteopathy may reduce non-specific neck and low back pain, and improve patients' quality of life (QoL) compared to control groups [16,17], and that manual therapy can reduce shoulder pain [18,19] and symptoms of knee and hip osteoarthritis [20]. Initial data from RCTs indicate that for acupuncture and osteopathy modest clinical benefits are achievable for a relatively small additional cost [21,22].

It is therefore timely and appropriate to consider how osteopathy and acupuncture treatments could effectively be provided by the NHS to treat pain. A number of general evaluations have demonstrated favourable results for the provision of complementary and alternative medicine (CAM) within the NHS [e.g. [4,23-27]]. However, the specific effectiveness (i.e. how well interventions work in the real world [28]) of providing osteopathy and acupuncture for MSK problems in the NHS has yet to be determined. Evaluation is needed to determine how to deliver these treatments effectively, and assess clinical outcomes and acceptability to patients and practices. The current paper reports on a service evaluation of an osteopathy and acupuncture service (delivered by complementary therapy practitioners) for patients with a range of MSK problems provided within a large GP practice in central London. The service design follows current guidelines which recommend the provision of manual therapy and acupuncture as a treatment option for persistent low back pain [6], and that GPs should act as gatekeepers to CAM services [29]. The evaluation examines patient outcomes, patient satisfaction, acceptability to stakeholders and the mode of provision (i.e. within general practice).

## Methods

### The osteopathy and acupuncture service

The service was based at the Victoria Medical Centre, a large GP practice in central London with approximately 11 500 registered patients. The practice serves a broad demographic of patients in an area with diverse ethnicities, a high level of asylum seekers and refugees, and with wards at the extremes of the deprivation scale (i.e. affluent and deprived) [30]. The service operated from September 2009 until August 2010. Two osteopaths and one acupuncturist provided 20 hours a week of treatment time. The service provided GPs with additional treatment options for patients registered at the practice who presented with MSK pain at routine surgery appointments. GPs based their referral decision on guidelines provided to them by the service director (DP) on MSK conditions appropriate for osteopathic or acupuncture treatment, taking patient preference into account. Patients could receive up to 6 treatments. Appointment making was integrated into the practice's computer-based reception system, so that patients could book their sessions in the normal way via the practice reception (in person or by telephone). Decisions about patients' treatments were not constrained by any research protocol, but were delegated to the practitioners who were free to treat as they would in everyday practice. Inclusion criteria for referral to the service were that the patient was experiencing MSK pain, registered at the GP practice, and referred by their GP. Exclusion criteria were patients aged less than 18 years or displaying symptoms indicative of a serious underlying condition. See Appendix for key aspects of the service model

GPs and CAM practitioners involved in the service were provided with formal training regarding intake criteria and forms, and the service and its delivery, evaluation and its ethical dimensions. Training was delivered by the evaluation manager (DR) and the service director (DP). For CAM practitioners, training comprised two face-to-face sessions (supplemented with written training materials) totalling five hours. Owing to constraints on GP time, GP training comprised provision of written materials supplemented with a lunchtime training session.

### The evaluation

To conduct a service evaluation, data were collected from a variety of sources. Quantitative patient outcome data were collected using pre- and post- treatment patient questionnaires, using consecutive sampling. Patient experiences and opinions of the service were obtained using a post-treatment service survey, collecting predominantly qualitative data. Interviews with healthcare professionals involved in the service collected qualitative data regarding their views of the service. These mixed methodologies are recommended for this type of evaluation [31]. Ethics approval for the evaluation was obtained from the University of Westminster Ethics Committee. Informed written consent was collected from all study participants.

#### Patient Questionnaires

Participants were provided with their pre-treatment questionnaire by reception staff when they booked their first appointment, and their post-treatment questionnaire by their acupuncturist/osteopath at the end of their final session. Participants who did not attend their final session had their questionnaire posted to them by the researcher (AC). All participants were able to ask a researcher for help completing the questionnaires, enabling participants with low literacy or whose first language was not English to be included in the evaluation. Participant demographics (including age, gender and ethnicity) and previous CAM use were collected by the pre-treatment questionnaire. Patient questionnaire measures included:

MSK pain, which was measured using the Bournemouth questionnaire (BQ) core items [32]. The BQ was developed specifically for patients with MSK pain and has been shown to be reliable, valid and responsive to clinical change [e.g. [32]]. The BQ incorporates dimensions of the biopsychosocial model for MSK pain including levels of pain, interference with everyday tasks and social activities, anxiety, depression, the extent to which work affects their condition and coping ability. It comprises seven items scored from 0 to 10 which can then be summed to provide a total score ranging from 0 to 70. Higher scores indicate increased MSK problems.

Quality of Life (QoL), which was measured using the EuroQol-5D (EQ-5D) [33] a widely used, generic measure of health-related quality of life. It is quick and easy to complete and has been shown to be valid and reliable [34,35]. The first part comprises five items (measuring mobility, self-care, usual activities, pain and anxiety/depression) which are graded on three levels according to severity. Using the established algorithms for the UK [36], these items were translated directly into index scores, ranging from -0.59 (worst possible health state) to 1 (best possible state). The second part is a visual analogue scale (VAS) measuring overall health, anchored 0 (worst possible health state) to 100 (best possible health state).

Participants were further asked if they were using analgesics, and about areas where they experienced pain and work status. They were also asked to rate their general health and well-being, and physical activity levels on a five and six point Likert scale respectively.

#### Service Survey

Participants who completed their post-treatment questionnaire were also asked to complete a service survey. According to patient preference, the service survey was available to complete online or by hand. Both versions of the survey were identical. The survey comprised a combination of open-ended questions with space for participants to write answers, as well as "yes" / "no" closed response questions aimed at ascertaining participants' opinions and experiences of the service including: perceived benefits, satisfaction, problems, suggestions for improvement, continuing provision of the service, treatment by staff and future use of acupuncture/osteopathy.

#### Healthcare professional and CAM practitioner interviews

All healthcare professionals involved in the service (all seven GPs and the administration manager at the practice, and the three CAM practitioners) were invited to participate in an interview. Semi-structured interviews aimed to elicit participants' views on the service were conducted approximately five months into the service by AC. While questions and topics were on the interview schedule, there was flexibility to follow up issues raised by the interviewee. Topics included benefits of the service, problems encountered, helpfulness to patients, ease of incorporation and improvements to the service. Interviews lasted between 10 and 20 minutes. Ten of the interviews were recorded; one was documented using note taking at the request of the participant.

### Data management and analysis

Quantitative data were analysed using SPSS version 16. Statistical significance was set at the 5% level. To ensure a conservative analysis, Non-parametric tests [37,38] (Mann-Whitney-U, Wilcoxon Signed Rank, McNemar and Chi-square as appropriate) were used to compare the differences between those who did and did not return questionnaires on baseline variables. Non-parametric tests were further used to compare pre- and post- treatment variables including the BQ, EQ-5D, physical activity, analgesic use, and current work status. Percentage of participants experiencing a clinically significant improvement was determined by calculating the effect size for the BQ (raw change score divided by the standard deviation of the baseline scores). An effect size of 0.5 has been found to represent a clinically significant change for the BQ [39].

Qualitative data were collected from the service survey and the healthcare professional interviews. All data were analysed using a descriptive thematic analysis [40]. Service survey data were read and re-read, and input into the qualitative data analysis tool Weft QDA [41]. AC and DR independently developed a list of themes and compared coding frameworks to debate and arrive at a final coding list. AC investigated the themes across the data in detail, in order to code all the data. For the analysis of healthcare professional interviews, interviews revealed that there was very little variation in experiences and opinions of the service amongst health professionals. Thus, analysis involved AC repeatedly listening to all interview recordings and writing down key points that each participant was making along with transcribing only representative quotes illustrating key points [42]. Using these notes a list of key themes and illustrative quotes was then compiled relating to healthcare professionals opinions and experiences of the service. The key issues across both sets of data were assembled into themes in order to explain the data collected. Typical quotes are used to illustrate findings.

### Results

The results are presented in three sections. Firstly, participant characteristics (patients and healthcare professionals) and response rates are presented. The second section examines patient outcomes using quantitative data from patient questionnaires and qualitative data from the service survey. The final section examines acceptability of the service to patients and stakeholders, using data from the service survey and interviews with healthcare professionals.

### Participant characteristics and response rates

#### Patient data

All 147 patients referred to the service after 19^th ^October 2009 who completed their treatments before the end of June 2010 were eligible to participate in the evaluation. 21 patients did not attend sessions and three did not want to participate in the evaluation; therefore data were available for 123 patients. Participants had a mean age of 49.0 years (SD 9.5, range 22-83 years). Seventy-nine (64.2%) were female, and the majority of participants were White (51.2%) or Black/Afro-Caribbean (12.2%). Thirty-one (25.2%) participants had experienced their pain for over 6 months, and half (50.4%) had previously experienced a similar complaint. The most common places participants experienced pain were their lower backs (53.7%), shoulders (43.9%) and necks (37.4%); 79.7% were taking pain medication. Twenty percent had used CAM before, commonly acupuncture (8.1%) and osteopathy (4.1%). According to the EQ-5D subscale 60% of patients rated their anxiety and depression as moderately (46%) or extremely (14%) high.

Participants waited a mean of 15.9 days from referral to the service to their first appointment; 48 (32.5%) received acupuncture, 87 (59.3%) osteopathy and 12 (8.1%) a combination of the two. Participants completed a mean of 4.7 sessions (SD 2.4). Twenty-eight (22.8%) participants stopped attending sessions.

Of the 123 participants, complete patient questionnaire data sets (pre- and post- treatment) were available for 102 participants. Data were examined for differences between those who did not respond and those who responded to their follow-up questionnaire. The only statistically significant difference found between responders and non-responders (using a Mann-Whitney-U test) was regarding the length of time the participant had been experiencing current painful episode (p = 0.015) (those with more chronic pain were more likely to respond to the follow-up questionnaire).

105 participants who completed their follow-up questionnaire were sent a service survey; and 91 (87%) returned their completed service survey to the evaluation team. Data were examined for differences between those who did not respond and those who responded to the service survey. The only statistically significant difference found between responders and non-responders (using a Mann-Whitney-U test) was on pre-treatment BQ scores (p = 0.014) (those with greater severity of condition were less likely to respond to the survey).

#### Healthcare professionals

All healthcare professionals invited to participate in interviews took part. Seven (64%) were female, six (55%) were of White-British ethnicity, two (18%) were Asian-British, two (18%) were East Asian and one was mixed ethnicity. Participants had an average age of 44.5 years (SD 8.9).

### Patient Outcomes

Comparisons between pre- and post-treatment for the primary outcome measure, the BQ, revealed a highly statistically significant improvement in MSK problems, including BQ total score (p < 0.0001) and all seven subscales: pain (p < 0.0001), interference with daily activities (p < 0.0001), interference with social routine (p < 0.0001), anxiety (p < 0.0001), depression (p < 0.0001), effect of work on pain (p < 0.0001), and coping with pain (p < 0.0001), see Table 1. Applying the threshold of 0.5 for effect size, 52.9%, 95%CI [42.3%, 61.7%] of participants experienced a clinically significant reduction in their MSK pain.

**Table 1:** BQ total and sub-scales scores pre and post-treatment

	Pre-treatment Median (interquartile range)		Post-treatment Median (interquartile range)		z score	p-value
**BQ total score** **(range 0-70 ↑ = worse)**	**38.5**	**(25.0-50.2)**	**23.0**	**(10.0-40.0)**	**5.77**	**< 0.0001**
BQ subscales (range 1-10 ↑ = worse)						
Pain	7.0	(5.0-8.0)	4.0	(2.0-7.0)	-6.25	<0.0001
Interference with activities	6.0	(3.0-8.0)	3.0	(1.0-7.0)	-5.23	<0.0001
Interference with social	5.0	(2.0-8.0)	2.5	(0.0-6.0)	-4.79	<0.0001
Anxiety	6.0	(3.0-8.0)	4.0	(1.0-7.0)	-4.57	<0.0001
Depression	4.0	(0.0-7.0)	3.0	(0.0-6.0)	-3.04	<0.0001
Effect of work	5.0	(2.0-8.0)	3.0	(1.0-7.0)	-3.61	<0.0001
Coping	5.0	(3.0-7.0)	3.0	(1.0-6.0)	-5.47	<0.0001

Comparisons between other study variables pre- and post-treatment revealed a statistically significant improvement in health-related QoL (EQ-5D index) (p < 0.0001), however, there was only a trend towards an improvement on the EQ-5D VAS (p = 0.064). A statistically significant reduction in analgesic use (82.8% to 68.7%, p < 0.003) was also found but there was no significant change for physical activity (p = 0.307) or general health and well-being (p = 0.541), see Table 2. There were inadequate numbers of participants in categories to conduct statistical analysis regarding current work status.

**Table 2:** Study variable scores pre and post-treatment

	Pre-treatment Median (interquartile range)		Post-treatment Median (interquartile range)		z-score	p-value
EQ-5D - index (range -.59-1 ↑ = better)	.440	(.137-.727)	.621	(.533-.796)	-4.82	< 0.0001
EQ-5D - VAS (range 0-100 ↑ = better)	70.0	(54.5-80.0)	70.0	(50.0-85.0)	-1.85	0.064
Physical activity (range 1-5 ↑ = better)	3.0	(2.0-4.0)	3.0	(2.0-4.0)	-1.02	0.307
Well-being and general health (range 1-6↑ = better)	3.0	(2.0-4.0)	3.0	(2.0-4.0)	-6.11	0.541

The service survey provided qualitative data supporting outcome benefits of the treatment to patients. In tune with the quantitative data, many patients reported that they valued the improvements in their MSK problem as a result of treatment. Patients reported decreased pain, and improved mobility including joint mobility. Some patients felt these improvements helped them to get on better with their daily lives.

"The treatment was really efficient. Since then I haven't had any problems with my back." P102

In addition, some patients reported improvements in other physical health conditions, for example decreased headaches, menstruation pain and improved energy levels. Other patients felt they had experienced improvements in their psychological well-being. Some patients described finding treatment relaxing and enjoyable, others experienced a reduction in their depression and anxiety, or felt more able to cope with their lives.

"It was surprisingly effective for many ailments. I had acupuncture and it helped with not only back pain but also illness reduction and depression." P84

Some patients felt better able to self-manage their condition. They learnt from practitioners a better understanding of their MSK problem, including what had caused it, what exacerbated it, and which exercises, stretches and changes to undertake to manage their condition better and prevent relapse.

"I was able to find out and understand more about what was wrong and learn new techniques to help me deal with the problem in my knees." P7

### Acceptability of the service

Service survey data showed that patient satisfaction with the service was extremely high. More than 9 in 10 participants reported that they were satisfied with the way they had been treated by staff in relation to the service, and 96.7% believed that the surgery should continue to provide the service in the future. Themes emerging from the qualitative analysis revealed the aspects of the service patients valued. Patients appreciated having the service at their GP practice, it was a convenient location and a familiar environment. They trusted a service provided through their GP practice, and felt reassured that their GP would know details about their osteopathy/acupuncture treatment. Patients also described finding the service straightforward (especially in terms of booking appointments), they appreciated the short waiting time for appointments and the efficient time-keeping of the service. In addition, some participants welcomed being offered a CAM therapy in the first place. They liked using these approaches compared to medication, as well as the additional time and attention given to the pain problem. Some patients liked the alternative (e.g. Chinese) explanatory model for health given by their practitioner, and the way in which treatment sought to get to the "root" of their problem. Other participants were just grateful that they had been offered something new to try to help with their MSK problem.

"Treatment at the practice would be in a familiar place and my doctor would be informed sooner than going to hospital and waiting. It's a good system, because you are being treated within your doctor's practice and communication should be more efficient. Local, to save you travelling to different hospitals." P43

"Osteopathy is an essential treatment as it treats the condition causing the pain. This is much better than taking painkillers." P25

A number of participants mentioned the positive qualities of their CAM practitioner. They valued the relationship they had formed with them, their professionalism and caring nature, and being provided with an explanation of the treatment they were receiving.

"The osteopath was very professional, pleasant and easy to talk to in regard to my problem. It was the first time I had been referred to an osteopath before and he was understanding and made me feel relaxed when being treated." P82

Ninety-one percent of participants said that they would use osteopathy/acupuncture again at their GP surgery, predominantly because they felt it had the potential to help MSK problems. This figure fell to only 30.8% who would use it privately, this was principally because of the cost of treatment, but also for the aforementioned reasons (e.g. convenience).

"Acupuncture and osteopathy are very good for people who suffer from pain, but in private it's very expensive. Myself I cannot afford to pay for it privately." P10

One quarter of participants said they had experienced some problems with the service. The analysis showed that the majority of these issues were related to the popularity of the service. For example, as the service became full, some participants had to wait for their first appointment, or for longer between appointments. Some participants wanted more appointment availability and flexibility (such as outside of working hours); others wanted to receive more and/or longer sessions. In addition, a small number of participants said they would like to receive more assistance from the reception, and some would have preferred a female practitioner.

"I had difficulty booking a time that would fit into my work schedule. Plus I couldn't book weekly appointments and I feel this was important for treatment. More flexible appointment times [needed]." P3

Interviews with healthcare professionals involved in the service also revealed high levels of satisfaction with the service among staff. In terms of service provision by the practice, all practitioners reported that the service had been incorporated well; overall the referral process had been simple and straightforward and the service ran smoothly. In relation to patient benefit, it was felt that the service was helpful for the practice's patients in terms of reducing their pain, increasing their flexibility and movement, improving general well-being, providing an explanation for their pain, and helping them to understand and manage their condition. In addition, GPs particularly valued having the service on site, this meant they were aware that their patients were having CAM treatment and were able to access details of patient appointments on the practice's computerised system and communicate with CAM practitioners easily. GPs also welcomed the relatively short waiting time for appointments and having an extra referral option.

"Very good, very prompt and the patients love it, you can't ask for more." GP4

"From referral to seeing patient, to appointment to getting feedback, I think it's worked very smoothly, there's been no logistical problem. It's very easy to do." GP3

Despite the favourable opinions of the service some problems emerged. Firstly, the popularity of the service needed to be managed. Interestingly, the service reached capacity very quickly. This high demand sometimes resulted in CAM practitioners being unable to treat their patients on a weekly basis, and there had been a period where GPs had been unable to refer to osteopathy. In addition, patients' expectations needed to be managed in terms of the total number of sessions they could receive. Secondly, CAM practitioners felt they could have benefited from more feedback regarding service provision from GPs and other members of staff.

*"If you see them *[*GPs*] *in the corridor they're very nice and say 'hi', but they're rushed off their feet, so there's virtually no time to have any interaction." Practitioner1*

Thirdly, there were issues regarding high numbers of referrals of chronic patients to the clinic. CAM practitioners were happy to try and treat any patient, but had to alter their expectations regarding the kind of success that was likely to be achieved with some patients.

*"Some *[*patients*] *have been very long-term and difficult, but I guess that's just the demographic you're just going to see here. From my point of view you just have to accept that and get on with it." Practitioner2*

"... we're so desperate to get some of these heart sink patients to be seen by somebody. And part of it is the therapy and part of it is the time they're spent with. And perhaps those types of patients were not quite so appropriate, but on the other hand you can't just pick up the easiest patients." GP1

Finally, there had initially been some practical problems regarding appropriate room space and equipment availability (e.g. couches) for practitioners. These issues had taken time to resolve and practitioners felt they should have been organised prior to their arrival.

In summary, data suggest that successful provision of osteopathy and acupuncture services for MSK pain within General Practice is achievable. However, some issues with provision will arise and need to be managed in order to provide as efficient a service as possible.

## Discussion

### Main findings

This study found that the provision of osteopathy and acupuncture treatment for MSK pain is achievable in a GP surgery with diverse patient demographics, complex MSK pain issues, high incidence of self-reported psychosocial problems, and minimal GP preparation for the CAM service. Despite a short service set-up time of a few months, patient and practice satisfaction with the CAM service was high, and patients with varied experiences of pain reported clinical improvement in their MSK condition and more (e.g. improved mental health and quality of life and a reduction in medication use). These findings are in line with NICE guidelines for the early management of persistent low back pain which recommend that treatment strategies should reduce pain and its impact on a person's life [6]. Our findings suggest that it is possible for a GP surgery to quickly adapt to incorporate a CAM pain service. However, certain conditions need to be in place, our qualitative results suggest, including adequate negotiation with practice staff, skilled CAM practitioners, built up demand for a pain service, appropriate rooms and equipment, adequate appointment flexibility, and minimal referral waiting times.

Furthermore, our results reveal patients are enthusiastic about the benefits of CAM treatments for pain when expertly delivered. Certainly, the importance of the patient being 'at the centre of everything the NHS does' is highlighted by the government [43]. However, the current study shows how high patient approval and demand for effective CAM services can have unexpected results. One drawback of the service was that patients wanted *more *CAM provision than originally estimated. Ideally there should be a degree of flexibility of CAM therapists to provide more or less appointments depending on patient demand.

Our results are in line with more general evaluations of CAM provision, for a variety of conditions, within the NHS which report statistically significant improvements in patients' specific conditions after treatment, improvement beyond symptom reduction (e.g. self-management behaviours, QoL), high levels of GP and patient satisfaction, reduced medication use, and that NHS provision allowed patients to access CAM services who would otherwise be unlikely to use such services [e.g. [4,23-27]]. They are also in line with other primary care based therapy-led service evaluations for MSK problems [e.g. [44]], which show such interventions are both feasible and acceptable to patients.

It is important to note the high levels of anxiety and depression among patients in this study. Anxiety and depression are common among chronic pain patients and can exacerbate pain [45-47], making mental health an important factor to address when treating these patients. BQ data showed patients in this study improved on all biopsychosocial dimensions of pain, including anxiety and depression. This suggests that holistic approaches like osteopathy and acupuncture may have added advantages for patient groups with relatively poor mental health. However, cognitive-behavioural and stress-management techniques may need to be considered alongside these therapies for patients who have psychological barriers to recovery [45,48,49].

### Strengths and limitations of the study

The results of this evaluation are important because osteopathy and acupuncture for MSK pain have not been evaluated together before, despite NICE guidelines recommending acupuncture and manual therapy as treatment options for patients with persistent low back pain. Whilst this evaluation does not address cause and effect, it provides important information for GPs considering a patient-centred osteopathy and acupuncture service for MSK problems. While it is always difficult to generalise from one specific GP practice to other practices, the site evaluated served a diverse population, suggesting that the results could have a degree of currency at different sites. In terms of limitations, it might be argued that some groups of patients were less well served by the service model provided (e.g. those who did not return surveys, those who did not respond as well to treatment). While this may well be true, patient questionnaires and clinical surveys had a patient response rate of 85% and 74% respectively, and few significant differences, demographically, between responders and non-responders. This suggests that we included a reasonably broad sample of respondents. In terms of ethnicity, our sample comprised 29% of patients from ethnic minorities; this is in contrast to 16% to 23% of ethnic minorities living in the wards that are served by the Victoria Medical Centre [50]. The additional percentage of ethnic minorities in our sample is predominantly accounted for by the number of Black/Afro-Caribbean participants; our sample comprised 12% Black/Afro-Caribbean participants in contrast to 4% to 6% living in the wards that are served by Victoria Medical Centre [50]. These figures suggest that ethnic minorities are well represented in this sample.

Given the number of statistical tests carried out, it is possible that type I errors may be evident. All patient outcomes that were statistically significant, however, remained significant after a Bonferroni correction was applied, hence the likelihood of type I errors is minimal.

GPs considered patient preference in their decision to refer to osteopathy or acupuncture, or another MSK service. This potentially resulted in patients with more positive attitudes towards acupuncture and osteopathy being referred. This may be considered a limitation of the study. However, the evaluation was intended to examine 'real world' conditions, in which GPs will consider patient preference when a number treatment options are available. Some patients saw the osteopath and acupuncturist, this was for a variety of reasons. Some patients were referred to one practitioner such as the osteopath and then went back to the GP to ask for further sessions with the acupuncturist and vice versa. On some occasions the osteopath referred the patient across to the acupuncturist and vice versa if they felt their colleague could treat the problem more effectively. Furthermore, as the demand for the service grew, having the referral route of both osteopathy and acupuncture was helpful for waiting list management.

When a new service is set up, waiting times can be artificially low as the service builds up to capacity. Although the evaluation did not commence until the third week of the service, it is likely that the waiting times at the beginning of evaluation do not reflect those recorded during the rest of the evaluation. Finally, it should be noted that although there were qualitative reports from patients of improved general health and well-being, quantitative data showed less change in this area, presumably because pain is not the only important factor to address for improved well-being [51].

### Implications for future research and clinical practice

With the new Coalition Government's health White Paper announcing that GPs will be assuming much of the commissioning responsibilities from PCTs, this study holds particular interest for commissioning consortia, as it demonstrates that it is possible to introduce treatment modalities into a GP surgery for patient benefit, even when the underlying philosophy (e.g. traditional Chinese medicine) differs to that of biomedicine. In addition, patients benefit from pain reduction and improvements in QoL, even after a relatively small number of treatments (less than 5). Nevertheless, flexibility of service provision does need to be considered. The experience of the service director (DP) suggests that there are a good number of suitably qualified osteopaths and acupuncturists available to work within the NHS.

This study reveals one mode of provision to be successful, but does not compare and contrast it with other modes, which may be more or less successful. Future research may wish to consider further the best way CAM services for MSK pain can be provided by the NHS and the extent to which these services should be integrated. For example, the service model examined by this study only represents CAM *provision *by the NHS. However, *integration *is not about merely providing CAM on the NHS; full integration of CAM services would involve conventional and CAM practitioners having a better understanding and appreciation of each others practices and working closely together for the benefit of the patient [52]. Future research may also wish to examine cost-effectiveness of osteopathy and acupuncture services, and longer-term patient outcomes to indicate the extent to which improvements are sustained.

## Conclusions

This study found statistically and clinically significant improvements in pain and quality of life among patients receiving acupuncture and osteopathy for their MSK pain, and high levels of patient and healthcare professional satisfaction with the service. This suggests that the provision of osteopathy and acupuncture for MSK pain is achievable in a GP surgery with diverse patient demographics, complex MSK pain issues (including high anxiety and depression) and minimal preparation for the CAM service. A GP surgery can quickly adapt to incorporate complementary therapy provided key principles are followed.

## Competing interests

The authors declare that they have no competing interests.

## Authors' contributions

DP designed and oversaw the delivery of the integrated service, DR designed the evaluation with input from MP. AC, DP and MP contributed to study implementation, and data analysis and interpretation. AC, DR and MP wrote the manuscript, DP contributed to aspects of the writing of the manuscript. All authors read and approved the final manuscript.

## Appendix 

• Service charitably funded for one year

• GP partners consulted on service requirements

• Need at GP practice for MSK services, in terms of services on offer and shorter waiting times than were available for the physiotherapy service

• Lead GP on Steering Group

• Recruitment of experiences CAM practitioners

• GPs completed referral forms stored on main Practice server

• Regular meetings between CAM practitioners

• CAM practitioners recorded clinical session notes on computerised EMIS system used by the whole Practice team

• Action research elements of the evaluation fed back into service delivery

## Pre-publication history

The pre-publication history for this paper can be accessed here:

http://www.biomedcentral.com/1471-2296/12/49/prepub
